# Distorted time window for sensorimotor integration and preserved time window for sense of agency in patients with post-stroke limb apraxia

**DOI:** 10.3389/fnhum.2025.1597200

**Published:** 2025-06-13

**Authors:** Satoshi Nobusako, Rintaro Ishibashi, Takaki Maeda, Sotaro Shimada, Shu Morioka

**Affiliations:** ^1^Neurorehabilitation Research Center, Kio University, Nara, Japan; ^2^Graduate School of Health Science, Kio University, Nara, Japan; ^3^Department of Rehabilitation, Murata Hospital, Osaka, Japan; ^4^Department of Neuropsychiatry, Keio University School of Medicine, Tokyo, Japan; ^5^Department of Electronics and Bioinformatics, School of Science and Technology, Meiji University, Kawasaki, Japan

**Keywords:** agency attribution task, apraxia, comparator model, delay detection task, sense of agency, sensorimotor integration, sensory-sensory integration

## Abstract

Limb apraxia is a cognitive-motor disorder typically resulting from left hemisphere stroke, characterized by an inability to perform skilled limb movements despite intact motor and sensory functions. Previous studies suggest that individuals with apraxia exhibit deficits in sensorimotor integration, particularly in detecting temporal discrepancies between movement and sensory feedback. However, whether these deficits affect explicit sense of agency (SoA) remains unclear. This study investigated the time window for sensorimotor integration and explicit SoA in post-stroke patients with and without apraxia. Twenty patients with left hemisphere stroke participated in a delay detection task assessing sensory-sensory and sensorimotor integration and an agency attribution task measuring explicit SoA. The results demonstrated that apraxic patients had a significantly prolonged delay detection threshold and reduced steepness in the active movement condition, indicating an altered time window for sensorimotor integration. In contrast, there were no significant differences between apraxic and non-apraxic patients in the time window for explicit SoA. These findings suggest that while apraxic patients exhibit deficits in sensorimotor integration, their explicit SoA remains preserved. This dissociation supports the notion that explicit SoA may be maintained through compensatory cognitive processes despite impairments at the sensorimotor level. Further research is needed, considering the limitations of this study, to achieve a more comprehensive understanding of SoA in apraxia.

## 1 Introduction

Limb apraxia is a condition typically associated with left hemispheric damage, characterized by an inability to perform skilled or learned limb movements upon request or imitation. This impairment occurs independently of sensory-motor deficits or cognitive impairments that hinder task comprehension or stimulus processing (Osiurak and Rossetti, 2017). Limb apraxia is generally considered to result from impairments in stored action representations (Garcea et al., 2020) and technical reasoning (Osiurak et al., 2021). However, studies have also demonstrated that deficits in sensorimotor integration, which serve as the foundation for these functions, are present in individuals with limb apraxia (Mutha and Haaland, 2014; Mutha et al., 2010; Nobusako et al., 2018).

Nobusako et al. (2018) demonstrated that patients with apraxia following brain injury exhibit impairments in detecting temporal congruency or incongruency between movement and its sensory feedback. Specifically, while these patients retained the ability to detect delayed visual feedback relative to tactile stimuli and passive movement (proprioception), they showed a significant impairment in detecting delayed visual feedback during active movement. Given that the time window for sensory-sensory integration remained intact, whereas the time window for motor-sensory integration was distorted, these findings suggested that individuals with apraxia may have deficits in motor prediction during self-generated movements, such as impairments in efference copy and predicted sensory feedback (corollary discharge).

On the other hand, the detection of temporal congruency or incongruency between an action and its outcome plays a crucial role in the generation of the Sense of Agency (SoA). SoA is the subjective experience of being the initiator and controller of one’s own actions, which is a basic and constant feature of human interactions with the external world (Gallagher, 2000; Synofzik et al., 2008a; Synofzik et al., 2008b; Synofzik et al., 2013). This phenomenon is supported by the central monitoring theory (i.e., the “comparator model”) (Blakemore et al., 2002; Frith et al., 2000a; Frith et al., 2000b; Wolpert et al., 1995). When a predicted action outcome matches its actual consequence, the outcome is more likely to be experienced as self-generated. Conversely, when a discrepancy occurs, the outcome is more likely to be perceived as externally generated (Frith et al., 2000a; Frith et al., 2000b). Indeed, numerous previous studies have demonstrated that a reduction in spatial or temporal mismatches (prediction errors) between an action and its outcome enhances SoA, whereas an increase in such mismatches leads to a diminished SoA (Asai and Tanno, 2007; David et al., 2007; David et al., 2011; Farrer and Frith, 2002; Farrer et al., 2003; Farrer et al., 2008a, 2008b; Franck et al., 2001; Kalckert and Ehrsson, 2012; Leube et al., 2003a, 2003b; Ratcliffe and Newport, 2017). Osumi et al. (2019) reported a significant correlation between the time window for detecting delayed visual feedback during active movement and the time window for SoA—the temporal interval between an action and its outcome within which SoA is maintained—in healthy individuals. This finding suggests a close relationship between sensorimotor integration and the SoA.

Therefore, given that patients with apraxia exhibit impairments in detecting the time window for temporal congruency or incongruency between movement and its sensory feedback, it is plausible to hypothesize that their time window for SoA may also be affected. Several researchers have suggested that apraxic patients may experience a disrupted sense of agency (SoA) (de Jong, 2011; Pazzaglia and Galli, 2014; Osiurak et al., 2019). Indeed, Wolpe et al. (2014) demonstrated a significant correlation between apraxia severity and diminished SoA, as measured by implicit indicators, in patients with corticobasal syndrome. However, no study to date has comprehensively examined whether this alteration in SoA involves both sensorimotor and cognitive components. To address this gap, the present study aimed to investigate whether patients with apraxia show changes not only in the time window for sensory-sensory and motor-sensory integration, but also in the time window for explicit SoA. We employed a delayed visual feedback detection task for passive and active movements to evaluate the temporal integration of sensory and motor signals, and an agency attribution task to assess explicit SoA. In doing so, we aimed to confirm the previously reported alteration in the time window for motor-sensory integration in patients with apraxia (Nobusako et al., 2018), and to clarify whether such alteration also leads to changes in the time window for explicit SoA in these patients.

In general, two types of tasks are used to investigate the sense of agency (SoA) (Haggard, 2006). Implicit SoA is typically assessed using the intentional binding effect, which refers to the subjective temporal compression between a voluntary action and its sensory outcome (Haggard et al., 2002; Haggard, 2006). In contrast, the agency attribution task is an explicit measure of SoA in which participants verbally report the extent to which they feel a sense of control over their own body or external events (Maeda et al., 2012, 2013; Maeda, 2019). In the present study, the delay detection task was used to assess the time windows for sensory-sensory and motor-sensory integration, and this task also required participants to explicitly report whether the visual feedback of their hand movement was delayed. Thus, this task primarily evaluates SoA-related processes at the explicit level. Accordingly, to examine the time window for SoA in a manner consistent with the delay detection task, we employed the agency attribution task, which similarly relies on verbal reports and reflects explicit awareness of agency. This consistency in measurement level allows for a coherent interpretation of the relationship between sensorimotor integration and explicit SoA.

## 2 Materials and methods

### 2.1 Participants

The participants were recruited from among patients receiving treatment and rehabilitation at Murata Hospital (Osaka, Japan). The inclusion criterion was the occurrence of left hemispheric stroke. The exclusion criteria were a history of a mental disorder or developmental disability, a cognitive disorder (a cut-off score of 21 or lower on the Mini Mental State Examination [MMSE]), impaired language comprehension precluding the understanding of how to perform the experimental task, or impaired field of vision.

In consideration of the frequent occurrence of apraxia and aphasia (Goldenberg and Randerath, 2015), we evaluated the presence of aphasia in the patients using the standard language test of aphasia (SLTA) (Japan Society for Higher Brain Dysfunction, 2003) and the supplementary tests for the SLTA (SLTA-ST) (Japan Society for Higher Brain Dysfunction, 2011) to be certain that the patients could understand and respond to the experimental task. The current study included patients whose SLTA Listening Comprehension items were at level 5 or 6, indicating no impairment in listening comprehension, and whose SLTA-ST Yes-No response items had a 100% correct response rate, i.e., no aphasia. In addition, this study included patients without motor and sensory deficits and without asomatognosia and somatoparaphrenia of the left upper extremity, the nonparalyzed side, as assessed by the Stroke Impairment Assessment Set (SIAS) (Liu et al., 2002) and the Verbal Asomatognosia and Somatoparaphrenia Assessment (Feinberg et al., 1990).

As a result, 20 patients with left hemispheric stroke (average age ± standard deviation [SD] of 67 ± 11.2 years, male = 8, all right-handed) consented to participate in the present study. Of the participating patients, those below the cutoff point as assessed by the Test of Upper Limb Apraxia (TULIA [AST]) (Vanbellingen et al., 2010; Vanbellingen et al., 2011) were considered in the apraxia group, and those above the cutoff point in the non-apraxia group. Originally, our study team aimed to enroll an equal number of participants in both groups (i.e., 11 patients per group). While 11 eligible non-apraxic patients consented to participate within the recruitment period, we continued recruiting apraxic patients until the apraxia group also reached 11 participants. However, over the course of one year, only 9 patients meeting the inclusion criteria for the apraxia group were available and consented to participate. Therefore, we finalized the sample size as 9 for the apraxia group and 11 for the non-apraxia group. Table 1 presents a summary of the demographic and clinical characteristics of the apraxia and non-apraxia groups (Supplementary material).

**Table 1:** Summary of information for the apraxia (*n* = 9) and non-apraxia (*n* = 11) groups.

Group		Age (years)	Sex	Handedness	Disease	Disease duration (days)	MMSE	Kohs	Apraxia			Left upper limb function	
									Imitation	Gesture	Total score	Motor function	Sensory function
Non-apraxia group (*n* = 11)	Mean	67.5	M, *n* = 4 F, *n* = 7	R, *n* = 11 L, *n* = 0	CI, *n* = 7 CH, *n* = 3 CT, *n* = 1	45.5	27.5	78.2	7.0	4.8	11.8	10.0	6.0
	SD	11.8				30.0	1.4	15.0	0.0	0.4	0.4	0.0	0.0
	Minimum	48				8	25	50	7	4	11	10	6
	Maximum	83				108	29	98.4	7	5	12	10	6
	Skewness	−0.307				0.805	−0.276	−0.353		−1.650	−1.650		
	Kurtosis	−1.257				−0.069	−1.584	−0.655		2.037	2.037		
Apraxia group (*n* = 9)	Mean	66.3	M, *n* = 4 F, *n* = 5	R, *n* = 9 L, *n* = 0	CI, *n* = 1 CH, *n* = 8 CT, *n* = 0	63.0	25.7	76.5	5.1	2.6	7.7	10.0	6.0
	SD	10.3				36.1	2.2	14.5	0.9	0.8	0.5	0.0	0.0
	Minimum	51				15	22	56.3	3	1	7	10	6
	Maximum	82				136	30	96	7	4	10	10	6
	Skewness	−0.288				0.582	0.434	0.106	−1.213	−0.177	−0.707		
	Kurtosis	−1.151				0.111	0.981	−1.615	3.281	0.144	−1.714		

All lesions are in the left hemisphere. CH, Cerebral hemorrhage; CI, Cerebral infarction; CT, Cerebral trauma; F, female; Kohs, Kohs block design Test; L, left; M, male; MMSE, mini mental state examination; R, right; SD, standard deviation.

The experimental procedure was approved by the research ethics committee of the affiliated institution (approval number: H27-16). There were no foreseeable risks to the participants, and no personally identifying information was collected. The participants provided background information and written informed consent. The procedures complied with the ethical standards of the Declaration of Helsinki regarding the treatment of human participants in research.

### 2.2 Procedures

Participating patients completed two experimental tasks: the delay detection task and the agency attribution task (Keio method). The order in which each task was performed was randomized. Because the delay detection task had two conditions, with each condition lasting no longer than 20 min, and the agency attribution task lasted no longer than 20 min, all participating patients completed the two experimental tasks within 60 min.

### 2.3 Delay detection task

**Figure 1:**
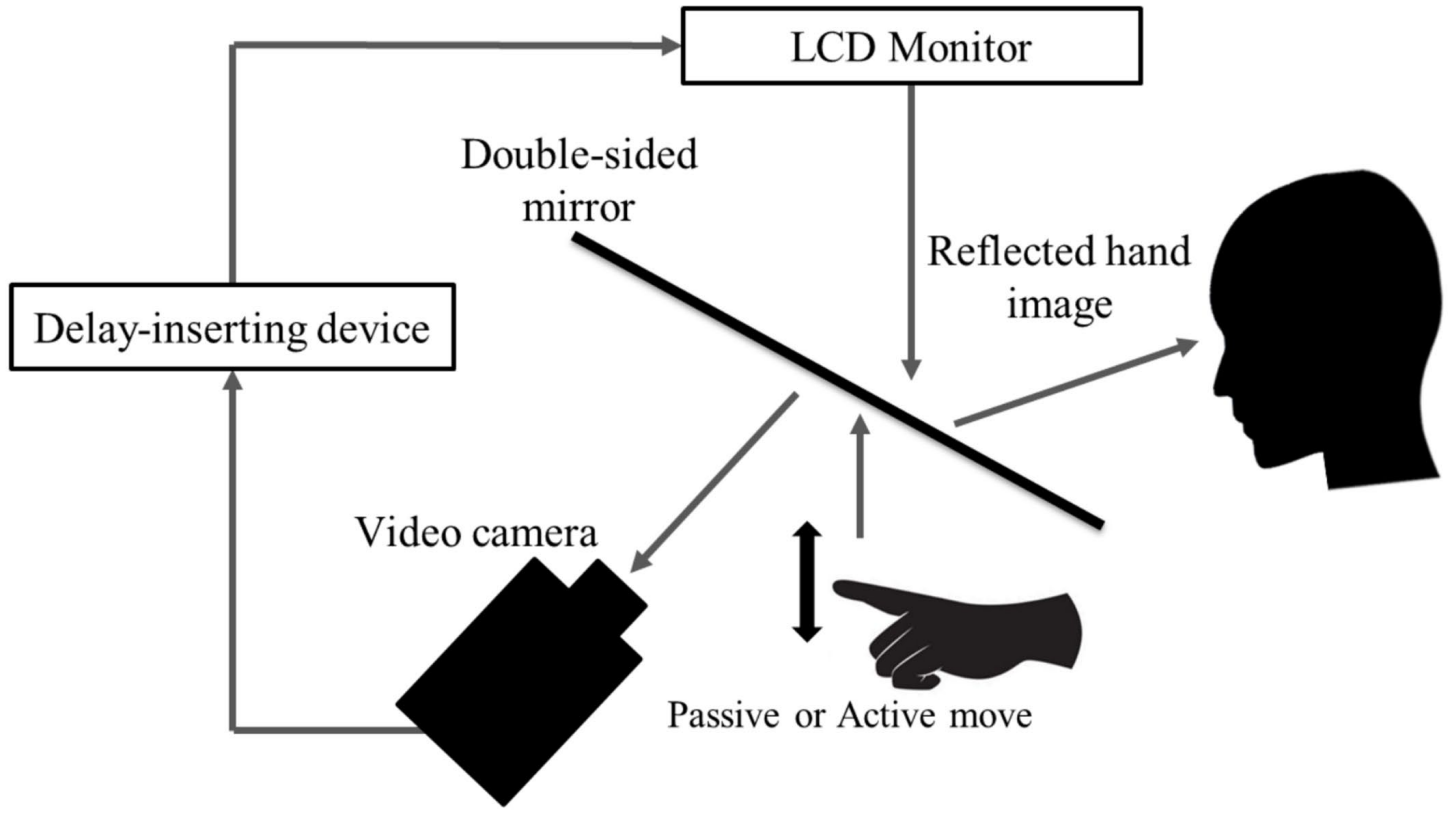
Delay detection task. The patient placed their left hand under a two-way mirror and the patient could see their left hand reflected in a two-way mirror. In the passive movement condition, the patient’s left index finger was moved passively; in the active movement condition, the patient moved their left index finger of their own volition. In both conditions, the fingers were captured by a video camera and the visual feedback delay was achieved using a hardware device. The patient observed the reflected image of their delayed finger displayed on an LCD monitor. For each trial of each stimulation condition, the patient was instructed to reply orally “delayed” or “not delayed” by the forced-choice method immediately following the trial.

We used a visual feedback delay detection task to quantify participants’ time window for delay detection (time window for sensory-motor integration) (Figure 1). The present experimental setup could systematically delay the time between movement execution and visual feedback, with an experimental system similar to that used in previous studies (Shimada et al., 2010; Nobusako et al., 2018; Osumi et al., 2019). Participants were not able to directly view their hand, which was placed under a double-sided tilted mirror. The reflected image of their hand in the double-sided mirror was filmed with a video camera (FDR-AXP35, Sony, Tokyo, Japan). The filmed hand image was sent to a liquid-crystal display monitor (LMD-A240, Sony) through a video delay device (EDS-3306, FOR-A YEM ELETEX, Tokyo, Japan). Finally, the hand images from the monitor were projected onto the double-sided mirror, enabling the participants to observe the image of their own hand reflected in the mirror without seeing their actual hand. The angle of the mirror was finely adjusted before the experiment, so that the reflected hand image was viewed from the participant’s perspective as if it were placed horizontally on the table. Visual feedback delay was introduced using a hardware device (EDS3305, ELETEX, Osaka, Japan) connected between the video camera and the monitor. Seven delay conditions (0, 100, 200, 300, 400, 500, and 600 msec delay) were tested. The intrinsic delay of the visual feedback in this experimental setting was approximately 33.71 msec, as measured by a time lag check device (EDD-5200, FOR-A YEM ELETEX, Tokyo, Japan).

All patients performed a delayed visual feedback detection task with their left hand in two stimulus conditions (passive and active) and seven delay conditions (0–600 ms). In the passive movement condition, passive extension (rising) and flexion (lowering) movements (movement of the index finger away from the supporting surface and returning back again to the supporting surface) were performed on the patient’s index finger. The passive extension (rising)-flexion (lowering) movements were performed by the experimenter raising and lowering a rod fixed to a hook and loop fastener (e.g., Velcro^®^) on the patient’s index finger. In the active movement condition, extension (raising)-flexion (lowering) movements of the index finger were performed based on each patient’s own volition. The patient was able to start the movement according to their own volition after the experimenter had informed them orally of the start of a trial. The patients had to answer orally whether or not there was a visual feedback delay compared to their own hand sensation/movement, in a forced-choice manner, immediately after the trial. The seven delay conditions of one trial were treated as one set, and seven sets were performed for each stimulation condition. The presentation order of the delay conditions in a single set was randomized. In addition, the order of the two stimulation conditions was also randomized across patients. Two stimulation conditions × 7 delay conditions × 7 sets were conducted for each patient’s left hand, resulting in a total of 98 trials. A 10 s rest period was set between each trial. In addition, a 3-min break period was set between each stimulation condition.

Logistic curves were fitted to the patient’s response in each stimulus condition according to the following equation (Shimada et al., 2010; Nobusako et al., 2018; Osumi et al., 2019):


$$P(t)=\frac{1}{1+\exp(-a(t-t_{\mathrm{DDT}}))}$$

where t was the visual feedback delay length, P(t) was the probability of delay detection, a indicated the steepness of the fitted curve, and tDDT indicated the observer’s DDT, representing the delay length at which synchrony and asynchrony judgment probabilities were equal (50%). In our experiment, t served as an independent variable, and P(t) was the observed value. Fitting was performed using a nonlinear least squares method (a trust-region algorithm), provided by the Curve Fitting toolbox in MATLAB R2016a (MathWorks, Natick, MA, United States), to estimate a (signifying the steepness of the logistic curve) and tDDT. DDT of the delay detection probability curve in the passive condition (passive-DDT) represents the time window for detecting delayed visual feedback to proprioception (passive movement) (i.e., the time window for sensory-sensory integration), and the steepness of the delay detection probability curve in the passive condition (passive-steepness) represents the clarity of delayed detection in passive conditions. DDT of the delay detection probability curve in the active condition (active-DDT) represents the time window for detecting delayed visual feedback to active movement (i.e., the time window for sensory-motor integration), and the steepness of the delay detection probability curve in the active condition (active-steepness) represents the clarity of delayed detection in active conditions.

### 2.4 Agency attribution task (Keio method)

**Figure 2:**
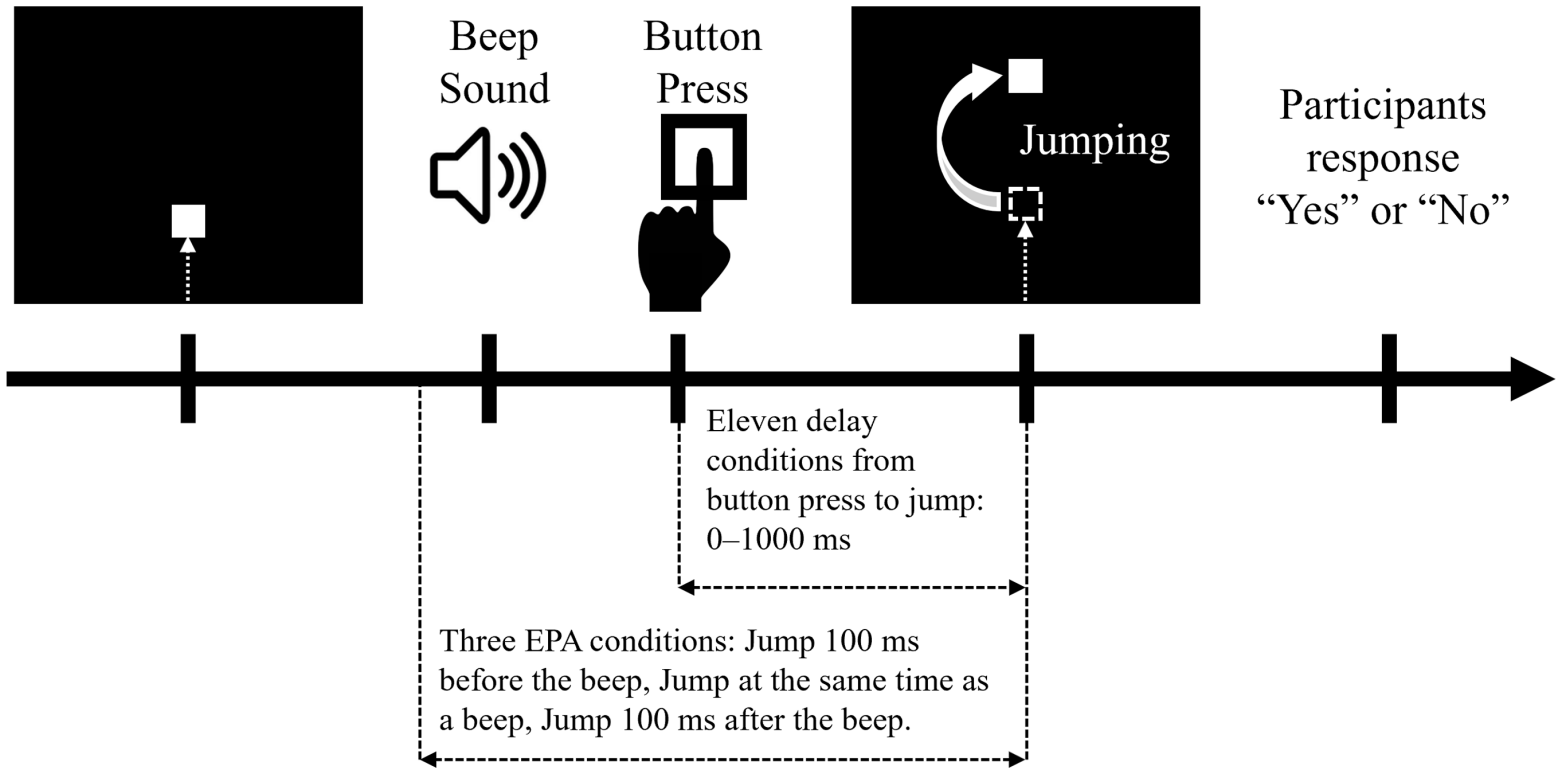
Agency attribution task. Each trial started with a dark computer screen. A square shape then appeared at the bottom of the screen and moved straight upward at a uniform speed (22 mm/s). The participants were instructed to press a button with their left index finger when they heard a beep. When the participant pressed a button, the square on the monitor jumped 35 mm upward, with various temporal biases. The jump of the square had action-linked conditions and event prior to action (EPA) conditions. In the action-linked conditions, temporal delays were introduced from 0 to 1,000 ms in 100-ms increments. EPA conditions were included in which the movement of the square on the screen was based on the beep and not on the button press, where the movement of the square was programmed to precede the participants’ intentional actions. There were three EPA conditions: the piece jumped at 100 ms before the beep, at the time of the beep, or at 100 ms after the beep. The participants answered “Yes” or “No” as to whether they felt that the square jumped as they intended.

An agency attribution task (Maeda et al., 2012, 2013; Maeda, 2019; Nobusako et al., 2020a,b; Nobusako et al., 2024; Osumi et al., 2019) was conducted to quantify the time window for SoA judgement in each participant (Figure 2). The experimental stimulus was presented on a 14-inch computer monitor. A 5-mm square shape appeared from the bottom of the screen and moved straight upward at a uniform speed (22 mm/s). The participants were instructed to push a key as quickly as possible with their left hand index finger when they heard a beep. After the participants pushed the Button, the square jumped 35 mm upward after a random delay (i.e., 0, 100, 200, 300, 400, 500, 600, 700, 800, 900, or 1,000 ms). Then, the participants were instructed to respond orally whether they felt that they had caused the square to jump upward as intended by giving a “Yes” or “No” response. A “Yes” response meant that the participants attributed the jump of the square to their Button press, i.e., they felt an SoA during the action. In this task, the participants were not asked to detect delayed visual feedback, but were asked to report an SoA to visual feedback (the jump of the square). In addition to these trials, “event prior to action” (EPA) trials were included in which the square jumped when the beep occurred instead of when the button was pressed (Maeda et al., 2012, 2013; Nobusako et al., 2020a,b; Nobusako et al., 2024; Osumi et al., 2019). The three EPA conditions were as follows: the square jumped at 100 ms before the beep, at the time of the beep, or at 100 ms after the beep. Under the EPA conditions, the participants responded verbally as to whether they felt the square had jumped as intended. There was no time limit between the jump of the square and the verbal response of the participant, and the next trial started after the participant responded. In accordance with previous study (Nobusako et al., 2020a,b; Nobusako et al., 2024), each delay/EPA condition was performed 5 times (i.e., 14 conditions × 5 times = 70 trials). The delay and EPA conditions were randomly mixed and executed. These procedures were completely consistent with previous studies (Maeda et al., 2012, 2013; Nobusako et al., 2020a,b; Nobusako et al., 2024; Osumi et al., 2019).

The probability of a “Yes” response for each delay condition (0, 100, 200, 300, 400, 500, 600, 700, 800, 900, or 1,000 ms), which did not involve response data in the EPA trials, was calculated for each participant. Positive “Yes” response probability values indicated a higher SoA in causing the square to jump. Logistic curves were fitted to the “Yes” response probability in the agency attribution task on the basis of the following formula (Nobusako et al., 2020a,b; Nobusako et al., 2024):


$$P(t)=\frac{1}{1+\exp(-a(t-t_{\mathrm{PSE}}))}$$

where t is the delay time, P(t) is the probability of a “Yes” response, a is the steepness of the fitted curve, and tPSE is the observer’s PSE, which demonstrates the delay time where “Yes” and “No” judgment probabilities are equal (50%). Fitting was performed by using a nonlinear least squares method (a trust-region algorithm) provided by the Curve Fitting toolbox in MATLAB R2016a (MathWorks, Natick, MA, United States). Therefore, the PSE was defined as the time window for SoA, and PSE represents the period of time in which SoA is maintained. In addition, the steepness (slope) of the “Yes” response probability curve was also calculated. The steepness of the curve indicates the sharpness of the contrast between the SoA judgments, and the higher the steepness, the clearer the SoA judgments.

### 2.5 Statistical analysis

Based on the group comparison result for one of the main outcomes, the DDT in the active condition (active-DDT), a *post hoc* power analysis was conducted using G*Power 3.1.9.7 (Faul et al., 2007, 2009). Statistical power ([1 − *β*]) was calculated using the effect size, *α* level, sample size, and number of groups.

The chi-square test for independence was used to compare sex between the apraxia and non-apraxia groups. The Shapiro–Wilk test showed that the age, disease duration, MMSE score, Kohs score, and active-DDT of the two groups were normally distributed, so an independent t-test was used to compare them.

The Shapiro–Wilk test showed that the DDT (passive-DDT) and the steepness (passive-steepness) of the delay detection probability curve in the passive condition, the steepness (active-steepness) of the delay detection probability curve in the active condition, and the PSE (the time window for SoA) and the steepness (SoA-steepness) of the SoA decision probability curve were not normally distributed in both groups. Therefore, the Mann–Whitney U test was used to compare them between the two groups. In addition, within-group comparisons were made between passive-DDT and active-DDT, and between passive-steepness and active-steepness for the apraxia and non-apraxia groups, respectively, using the Wilcoxon signed-rank test.

Finally, correlation analysis between the obtained data was performed using Pearson’s correlation coefficient or Spearman’s rank correlation coefficient.

We set the significance level at α = 0.05 for all analyses, and we used Bonferroni’s correction to adjust for multiple comparisons. In addition, we calculated the effect size.

The significance level was set at α = 0.05 for all analyses, and the effect size was also calculated. Bonferroni’s correction was used to adjust for multiple comparisons. All statistical analyses were performed using SPSS ver. 26 (IBM Corporation, Armonk, NY, United States).

## 3 Results

Using the effect size derived from the group comparison of active-DDT (d = 1.72), a sample size of 11 in the non-apraxia group and 9 in the apraxia group, and an α level of 0.05, a post hoc power analysis was performed using G*Power 3.1.9.7. The analysis revealed a statistical power ([1 − β]) of 0.9511108.

There were no significant differences in age (*t*(18) = 0.229, *p* = 0.821, *r* = 0.05, *d* = 0.11), sex (*χ2*(1) = 0.135, *p* = 0.714, *φ* = 0.082), disease duration (*t*(18) = −1.127, *p* = 0.275, *r* = 0.26, *d* = 0.529), MMSE score (*t*(18) = 2.098, *p* = 0.050, *r* = 0.44, *d* = 1.00), or Kohs score (*t*(18) = 0.248, *p* = 0.807, *r* = 0.06, *d* = 0.12) between the two groups. AST scores were significantly decreased in the apraxia group compared to the non-apraxia group (*z* = −4.010, *p* < 0.001, *r* = −0.897).

**Figure 3:**
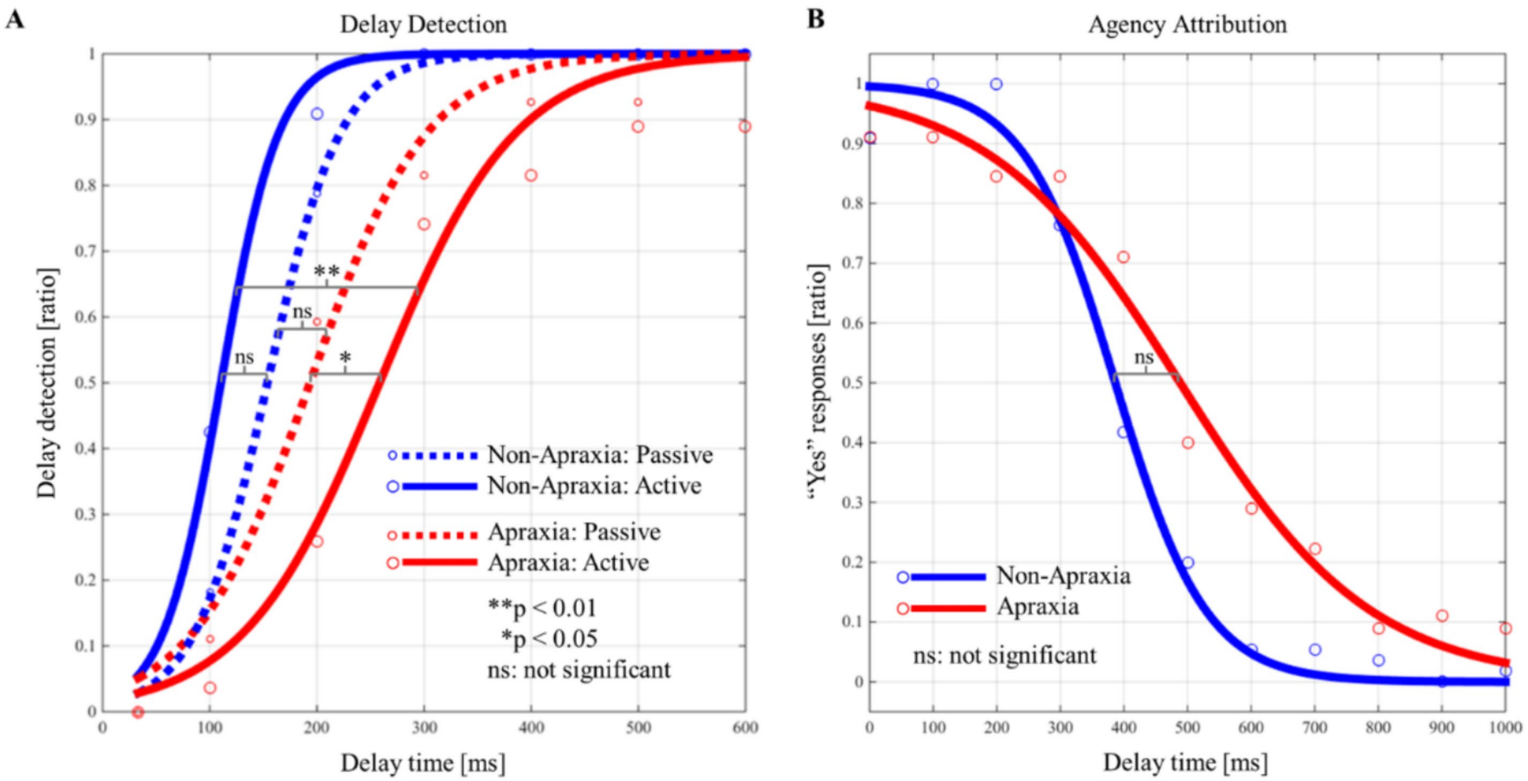
Probability curves for delay detection and the “Yes” responses (sense of agency) in the apraxia and non-apraxia groups. **(A)** Probability curves of the detection of the delay in the passive and active conditions for both groups. The horizontal axis represents the delay time (ms), and the vertical axis represents the probability of delay detection. The blue solid line indicates the probability curve for the active condition in the non-apraxia group; the red dotted line indicates the probability curve for the passive condition in the apraxia group; and the red solid line indicates the probability curve for the active condition in the apraxia group. Statistical significance indicated in the figure refers to within-group and between-group comparisons of the delay detection threshold. **p ≤ 0.01, *p ≤ 0.05, ns: not significant. **(B)** Probability curves of the “Yes” responses (sense of agency) in both groups. The horizontal axis represents the delay time (ms), and the vertical axis represents the probability of “Yes” responses. The blue solid line indicates the probability curve for the non-apraxia group, and the red solid line indicates the probability curve for the apraxia group. Statistical significance indicated in the figure refers to within-group and between-group comparisons of the point of subjective equality. **p ≤ 0.01, *p ≤ 0.05, ns: not significant.

**Figure 4:**
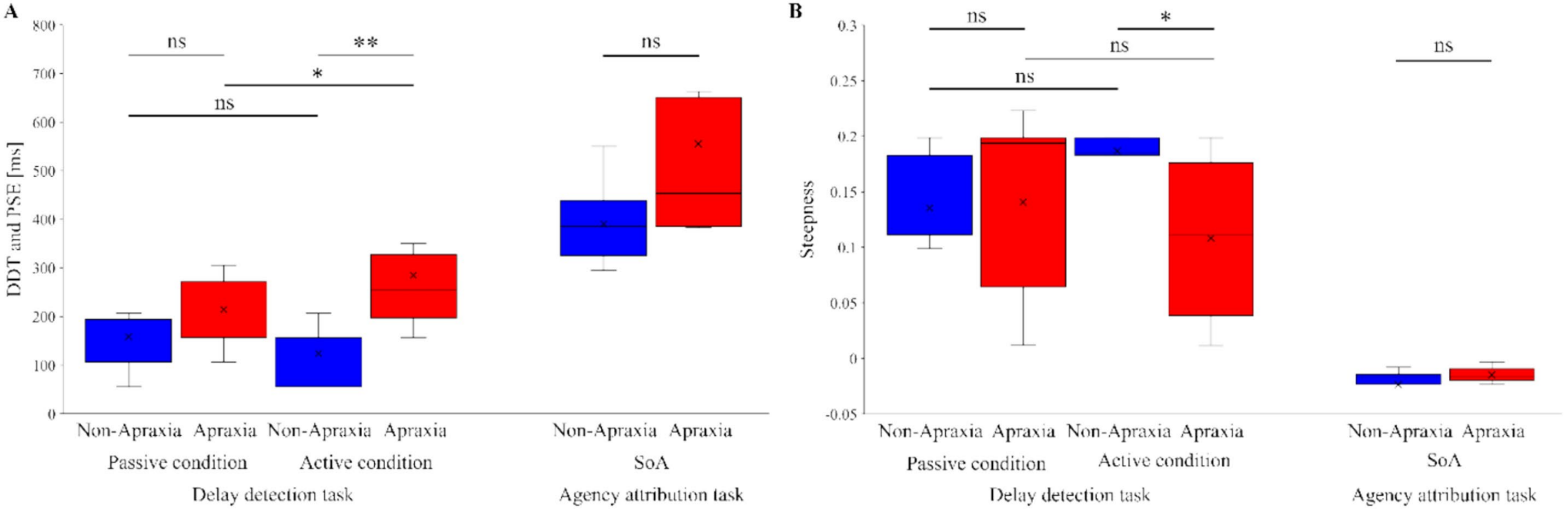
Results of inter- and intra-group comparisons of delay detection and agency attribution task data. **(A)** Results of between-group and within-group comparisons of the delay detection threshold (DDT) in the passive and active conditions in the delay detection task, and the point of subjective equality (PSE) in the agency attribution task. The blue boxes represent the non-apraxia group, and the red boxes represent the apraxia group. Boxes represent the lower, median, and upper quartiles. Lines represent the range of the minimum and maximum. **p < 0.01, *p < 0.05, ns: not significant. **(B)** Results of between-group and within-group comparisons of the steepness in the delay detection probability curves and the sense of agency judgment probability curves. The blue boxes represent the non-apraxia group, and the red boxes represent the apraxia group. Boxes represent the lower, median, and upper quartiles. Lines represent the range of the minimum and maximum. **p < 0.01, *p < 0.05, ns: not significant.

Figure 3 shows the delay detection probability curves for the passive and active conditions (Figure 3A), and the SoA decision probability curves (Figure 3B), in both groups. In both groups, the performance data for the delay detection task fitted a logistic curve, showing an increase in the probability of delay detection with increasing delay time (Figure 3A). In both groups, the response data for the agency attribution task were fitted to a logistic curve, and the SoA showed a decrease with increasing delay time (Figure 3B). Figure 4 shows the results of the between-group and within-group comparisons of the data from the delay detection task and the agency attribution task (Figures 4A,B). There were no significant differences in passive-DDT (*z* = −0.653, *p* = 0.552, *r* = −0.146), passive-steepness (*z* = −1.037, *p* = 0.331, *r* = −0.232), PSE (*z* = −1.886, *p* = 0.067, *r* = −0.422), and SoA-steepness (*z* = −0.970, *p* = 0.370, *r* = −0.217) between the two groups, but active-DDT and active-steepness in the apraxia group were significantly prolonged and decreased, respectively, compared to those in the non-apraxia group (active-DDT, *t*(18) = −3.620, *p* = 0.002, *r* = 0.65, *d* = 1.72; active-steepness, *z* = −2.046, *p* = 0.041, *r* = −0.457). In the non-apraxia group, there were no significant differences between passive-DDT and active-DDT (z = −1.904, *p* = 0.057, r = −0.406), and between passive-steepness and active-steepness (z = −1.666, *p* = 0.096, r = −0.355). In the apraxia group, there was no significant difference between passive and active-steepness (z = −1.859, *p* = 0.063, r = −0.438), but active-DDT was significantly prolonged compared to passive-DDT (z = −2.380, *p* = 0.017, r = −0.561).

Table 2 shows the correlation matrix. Correlation analysis revealed significant correlations between AST scores and MMSE scores (rs = 0.471, *p* = 0.036), active-DDT (rs = −0.743, *p* < 0.001), and active-steepness (rs = 0.474, *p* = 0.040) (Table 2).

**Table 2:** Correlation matrix.

*n* = 20		AST	Age	Delay detection task				Agency attribution task		MMSE	Kohs
				Passive-DDT	Passive-steepness	Active-DDT	Active-steepness	PSE	SoA-steepness		
AST		ー									
Age		0.019	ー								
Delay detection task	Passive-DDT	−0.254	0.185	ー							
	Passive-steepness	−0.279	−0.516*	−0.312	ー						
	Active-DDT	−0.743**	0.302	0.766**	0.062	ー					
	Active-steepness	0.474*	0.070	0.080	−0.150	−0.595**	ー				
Agency attribution task	PSE	−0.383	−0.002	0.375	0.037	0.297	−0.008	ー			
	SoA-steepness	−0.211	0.339	0.389	−0.393	0.326	−0.122	0.333	ー		
MMSE		0.471*	−0.392	−0.397	0.072	−0.501*	0.365	−0.119	−0.399	ー	
Kohs		0.169	−0.589**	−0.423	0.305	−0.493*	0.336	−0.080	−0.491*	0.431	ー

**p < 0.01, *p < 0.05. Numbers in the table show correlation coefficients. AST, apraxia screen of TULIA (the test of upper limb apraxia); DDT, the delay detection threshold; Kohs, the Kohs block design test; MMSE, the mini mental state examination; PSE, the point of subjective equality; SoA, sense of agency.

## 4 Discussion

Consistent with previous findings (Nobusako et al., 2018), the present study demonstrated that in patients with apraxia, the detection of delayed visual feedback for passive movement (proprioception)—that is, the time window for sensory-sensory integration—was comparable to that of non-apraxic patients. However, the detection of delayed visual feedback for active movement—the time window for sensory-motor integration—was significantly impaired (i.e., prolonged and diminished) compared to non-apraxic patients. Furthermore, a significant correlation was observed between the severity of apraxia and the difficulty in detecting delayed visual feedback during active movement, as indicated by prolonged active-DDT and reduced active-steepness. However, there was no significant difference between apraxic and non-apraxic patients in the time window for SoA, as measured by the agency attribution task. These findings suggest that while patients with apraxia exhibit deficits at the sensorimotor level, which constitutes a fundamental component of SoA generation, their explicit SoA itself remains preserved.

Previous studies have shown that while patients with apraxia do not exhibit impairments in detecting delayed visual feedback for tactile stimuli or passive movement, they do have difficulty detecting delayed visual feedback during active movement (Nobusako et al., 2018). Consistent with these findings, the present study also demonstrated that apraxic patients showed no significant difference from non-apraxic patients in detecting delayed visual feedback for passive movement. However, their ability to detect delayed visual feedback during active movement was significantly impaired compared to non-apraxic patients. This impairment may be attributable to deficits in motor prediction mechanisms (e.g., efference copy and predicted sensory feedback [corollary discharge]) during self-generated movement in apraxic patients. Indeed, several previous studies employing motor imagery tasks (Buxbaum et al., 2005; Sirigu et al., 1996; Sirigu and Duhamel, 2001; Ochipa et al., 1997; Tomasino et al., 2003) and electroencephalography (EEG) (Sirigu et al., 2004; Fontana et al., 2012) have demonstrated that apraxic patients have difficulty predicting the sensory consequences of their own movements. Moreover, this notion is further supported by the findings of the present study, in which no significant correlation was observed between apraxia severity and the performance in the passive movement condition of the delay detection task. In contrast, a significant correlation was found between apraxia severity and the difficulty in detecting delayed visual feedback during active movement, reinforcing the idea that impaired motor prediction mechanisms underlie this deficit.

The dissociation observed in this study—namely, impaired performance in the delay detection task for active movement despite preserved explicit SoA—invites a more nuanced theoretical framing grounded in recent hierarchical models of the sense of agency (SoA). According to Synofzik et al. (2008a), SoA comprises multiple levels of processing: the feeling of agency (FoA), grounded in low-level sensorimotor cues; the judgment of agency (JoA), which incorporates higher-order cognitive processes such as beliefs, intentions, and expectations; and the ascription of responsibility (AoR), which operates within a socio-normative domain involving moral reasoning and abstract inferential processes. In parallel, recent schizophrenia research by Oi et al. (2024) proposed a tripartite model of SoA processing—motor control, control detection, and self-attribution—and demonstrated that despite deficits at the sensorimotor level, self-attribution may remain preserved through compensatory mechanisms.

Our findings align with these frameworks: patients with apraxia showed disturbances likely corresponding to FoA-level deficits (or impairments in motor control and control detection), as reflected in the degraded performance in the active condition of the delay detection task. However, their explicit SoA, presumably indexed by JoA or self-attribution, was comparable to that of non-apraxic patients. This pattern suggests that compensatory cognitive mechanisms—possibly involving intact metacognitive monitoring, top-down cognitive control, or the integration of contextual and conceptual information—may support the preservation of explicit agency judgment despite disrupted sensorimotor integration. These higher-order processes may “override” prediction error signals generated by sensorimotor anomalies, enabling individuals to maintain a coherent sense of authorship over actions at the conscious level. Future studies could benefit from directly examining such metacognitive or control-related processes to elucidate how the hierarchical SoA system maintains functional coherence under conditions of partial dysfunction.

This study has several limitations. First, although the sample size was relatively small (*n* = 20) and the severity of apraxia in the apraxic group was mild (mean AST score = 7.7), a *post hoc* power analysis based on the group difference in active-DDT revealed a sufficiently high statistical power (1 − *β* = 0.951). Thus, we consider the primary group comparison to be statistically robust. Nevertheless, the small sample size and mild symptom severity may still limit the generalizability of the findings to the broader population of individuals with more severe or heterogeneous presentations of apraxia.

Additionally, while the current study employed an agency attribution task to assess explicit SoA, previous research using implicit SoA paradigms (e.g., intentional binding; Wolpe et al., 2014) suggests that explicit and implicit dimensions of agency may dissociate. Therefore, future research should include larger samples, encompass patients with a wider range of apraxic severity, and utilize both explicit and implicit measures of SoA to further clarify the relationship between apraxia and agency.

Moreover, the current study did not incorporate objective instrumental assessments, such as neuroimaging, kinematic analysis, or electrophysiological measures, which could have provided more direct evidence of the proposed sensorimotor integration deficits. Furthermore, standardized clinical scales for upper limb function, such as the Fugl-Meyer Assessment or the Action Research Arm Test (ARAT), were not administered. These omissions may limit the translational applicability of the findings to functional motor outcomes in rehabilitation settings. Future studies should integrate these objective and clinically relevant assessments to validate and extend the current behavioral findings and to better inform targeted therapeutic strategies.

The findings obtained in this study also carry potential clinical implications. The dissociation between impaired sensorimotor integration and preserved explicit SoA in apraxia suggests that targeted rehabilitation strategies may harness higher-order cognitive mechanisms to support agency experience. For instance, interventions that emphasize cognitive-motor integration—such as action observation training (Pazzaglia and Galli, 2019; Pazzaglia and Galli, 2015) or combined action execution and observation protocols—may enhance sensorimotor prediction and recalibration. These approaches aim to strengthen the perceptual-motor coupling that underlies voluntary action, thereby compensating for impaired predictive signals. Moreover, recent work highlights the value of using multisensory stimulation (e.g., visual, auditory, proprioceptive cues) to facilitate motor relearning and plasticity, which could be particularly beneficial in patients with disrupted motor-sensory prediction loops (Pazzaglia, 2022; Galli et al., 2020). From this perspective, therapeutic techniques that incorporate predictive feedback modulation and perceptual recalibration may contribute to restoring a coherent experience of agency in individuals with apraxia, even in the presence of residual sensorimotor deficits.

## 5 Conclusion

This study demonstrated that in patients with post-stroke apraxia, while the time window for sensorimotor integration, which constitutes a fundamental component of SoA generation, was impaired, the time window for explicit SoA remained preserved. These findings provide important insights into the clinical management of apraxia. The dissociation between impaired sensorimotor integration and preserved explicit sense of agency suggests that higher-order cognitive mechanisms may compensate for sensorimotor deficits in these patients. This implies that cognitive-motor rehabilitation strategies—such as action observation training, multisensory stimulation, and interventions targeting predictive feedback—could enhance agency experience and functional recovery in apraxia. Future studies should systematically examine the efficacy of such interventions and investigate how explicit and implicit components of agency interact in diverse clinical populations with varying severity and types of apraxia.

## Data Availability

The raw data supporting the conclusions of this article will be made available by the authors, without undue reservation.

## References

[ref1] Asai T. Tanno Y. (2007). The relationship between the sense of self-agency and schizotypal personality traits. J. Mot. Behav. 39, 162–168. doi: 10.3200/JMBR.39.3.162-168, PMID: 17550868

[ref2] Blakemore S. J. Wolpert D. M. Frith C. D. (2002). Abnormalities in the awareness of action. Trends Cogn. Sci. 6, 237–242. doi: 10.1016/s1364-6613(02)01907-1, PMID: 12039604

[ref3] Buxbaum L. J. Johnson-Frey S. H. Bartlett-Williams M. (2005). Deficient internal models for planning hand-object interactions in apraxia. Neuropsychologia 43, 917–929. doi: 10.1016/j.neuropsychologia.2004.09.006, PMID: 15716162

[ref4] David N. Cohen M. X. Newen A. Bewernick B. H. Shah N. J. Fink G. R.. (2007). The extrastriate cortex distinguishes between the consequences of one’s own and others’ behavior. NeuroImage 36, 1004–1014. doi: 10.1016/j.neuroimage.2007.03.030, PMID: 17478105

[ref5] David N. Stenzel A. Schneider T. R. Engel A. K. (2011). The feeling of agency: empirical indicators for a pre-reflective level of action awareness. Front. Psychol. 2:149. doi: 10.3389/fpsyg.2011.00149, PMID: 21779268 PMC3133862

[ref6] de Jong B. M. (2011). Neurology of widely embedded free will. Cortex 47, 1160–1165. doi: 10.1016/j.cortex.2011.06.011, PMID: 21752362

[ref7] Farrer C. Bouchereau M. Jeannerod M. Franck N. (2008a). Effect of distorted visual feedback on the sense of agency. Behav. Neurol. 19, 53–57. doi: 10.1155/2008/425267, PMID: 18413918 PMC5452467

[ref8] Farrer C. Franck N. Georgieff N. Frith C. D. Decety J. Jeannerod M. (2003). Modulating the experience of agency: a positron emission tomography study. NeuroImage 18, 324–333. doi: 10.1016/s1053-8119(02)00041-1, PMID: 12595186

[ref9] Farrer C. Frey S. H. Van Horn J. D. Tunik E. Turk D. Inati S.. (2008b). The angular gyrus computes action awareness representations. Cereb. Cortex 18, 254–261. doi: 10.1093/cercor/bhm050, PMID: 17490989

[ref10] Farrer C. Frith C. D. (2002). Experiencing oneself vs another person as being the cause of an action: the neural correlates of the experience of agency. NeuroImage 15, 596–603. doi: 10.1006/nimg.2001.1009, PMID: 11848702

[ref9001] Faul F. Erdfelder E. Buchner A. Lang A. G. (2009). Statistical power analyses using G*Power 3.1: Tests for correlation and regression analyses. Behav. Res. Methods. 41, 1149–1160. doi: 10.3758/BRM.41.4.1149 19897823

[ref9002] Faul F. Erdfelder E. Lang A. G. Buchner A. (2007). G*Power 3: A flexible statistical power analysis program for the social, behavioral, and biomedical sciences. Behav. Res. Methods. 39, 175–191. doi: 10.3758/BF03193146 17695343

[ref11] Feinberg T. E. Haber L. D. Leeds N. E. (1990). Verbal asomatognosia. Neurology 40, 1391–1394. doi: 10.1212/wnl.40.9.1391, PMID: 2392224

[ref12] Fontana A. P. Kilner J. M. Rodrigues E. C. Joffily M. Nighoghossian N. Vargas C. D.. (2012). Role of the parietal cortex in predicting incoming actions. NeuroImage 59, 556–564. doi: 10.1016/j.neuroimage.2011.07.046, PMID: 21839178

[ref13] Franck N. Farrer C. Georgieff N. Marie-Cardine M. Daléry J. d’Amato T.. (2001). Defective recognition of one’s own actions in patients with schizophrenia. Am. J. Psychiatry 158, 454–459. doi: 10.1176/appi.ajp.158.3.454, PMID: 11229988

[ref14] Frith C. D. Blakemore S. Wolpert D. M. (2000a). Explaining the symptoms of schizophrenia: abnormalities in the awareness of action. Brain Res. Brain Res. Rev. 31, 357–363. doi: 10.1016/s0165-0173(99)00052-1, PMID: 10719163

[ref15] Frith C. D. Blakemore S. J. Wolpert D. M. (2000b). Abnormalities in the awareness and control of action. Philos. Trans. R. Soc. Lond. Ser. B Biol. Sci. 355, 1771–1788. doi: 10.1098/rstb.2000.0734, PMID: 11205340 PMC1692910

[ref16] Gallagher I. I. (2000). Philosophical conceptions of the self: implications for cognitive science. Trends Cogn. Sci. 4, 14–21. doi: 10.1016/s1364-6613(99)01417-5, PMID: 10637618

[ref17] Galli G. Cakmak Y. O. Babič J. Pazzaglia M. (2020). Editorial: embodying tool use: from cognition to neurorehabilitation. Front. Hum. Neurosci. 14:585670. doi: 10.3389/fnhum.2020.585670, PMID: 33192422 PMC7606925

[ref18] Garcea F. E. Greene C. Grafton S. T. Buxbaum L. J. (2020). Structural disconnection of the tool use network after left hemisphere stroke predicts limb apraxia severity. Cereb Cortex Commun. 1:tgaa035. doi: 10.1093/texcom/tgaa035, PMID: 33134927 PMC7573742

[ref19] Goldenberg G. Randerath J. (2015). Shared neural substrates of apraxia and aphasia. Neuropsychologia 75, 40–49. doi: 10.1016/j.neuropsychologia.2015.05.017, PMID: 26004063

[ref20] Haggard P. (2006). “Conscious intention and the sense of agency” in Disorders of volition. eds. Sebanz N. Prinz W. (Massachusetts: MIT Press), 175–192.

[ref21] Haggard P. Clark S. Kalogeras J. (2002). Voluntary action and conscious awareness. Nat. Neurosci. 5, 382–385. doi: 10.1038/nn827, PMID: 11896397

[ref22] Japan Society for Higher Brain Dysfunction (2003). The standard language test of aphasia (SLTA). Tokyo: Shinkoh-Igaku-Shuppansha.

[ref23] Japan Society for Higher Brain Dysfunction (2011). The supplementary tests for standard language test of aphasia (SLTA-ST). Tokyo: Shinkoh-Igaku-Shuppansha.

[ref24] Kalckert A. Ehrsson H. H. (2012). Moving a rubber hand that feels like your own: a dissociation of ownership and agency. Front. Hum. Neurosci. 6:40. doi: 10.3389/fnhum.2012.00040, PMID: 22435056 PMC3303087

[ref25] Leube D. T. Knoblich G. Erb M. Grodd W. Bartels M. Kircher T. T. (2003a). The neural correlates of perceiving one’s own movements. NeuroImage 20, 2084–2090. doi: 10.1016/j.neuroimage.2003.07.033, PMID: 14683712

[ref26] Leube D. T. Knoblich G. Erb M. Kircher T. T. J. (2003b). Observing one’s hand become anarchic: an fMRI study of action identification. Conscious. Cogn. 12, 597–608. doi: 10.1016/S1053-8100(03)00079-5, PMID: 14656503

[ref27] Liu M. Chino N. Tuji T. Masakado Y. Hase K. Kimura A. (2002). Psychometric properties of the stroke impairment assessment set (SIAS). Neurorehabil. Neural Repair 16, 339–351. doi: 10.1177/0888439002239279, PMID: 12462765

[ref28] Maeda T. (2019) Method and device for diagnosing schizophrenia. International application no. PCT/JP2016/087182 (Japanese patent no. 6560765, 2019). Japan Patent Office.

[ref29] Maeda T. Kato M. Muramatsu T. Iwashita S. Mimura M. Kashima H. (2012). Aberrant sense of agency in patients with schizophrenia: forward and backward over-attribution of temporal causality during intentional action. Psychiatry Res. 198, 1–6. doi: 10.1016/j.psychres.2011.10.021, PMID: 22374553

[ref30] Maeda T. Takahata K. Muramatsu T. Okimura T. Koreki A. Iwashita S.. (2013). Reduced sense of agency in chronic schizophrenia with predominant negative symptoms. Psychiatry Res. 209, 386–392. doi: 10.1016/j.psychres.2013.04.017, PMID: 23680465

[ref31] Mutha P. K. Haaland K. Y. (2014). Cognitive aspects of motor control. Cortex 57, 299–300. doi: 10.1016/j.cortex.2014.03.001, PMID: 24726068

[ref32] Mutha P. K. Sainburg R. L. Haaland K. Y. (2010). Coordination deficits in ideomotor apraxia during visually targeted reaching reflect impaired visuomotor transformations. Neuropsychologia 48, 3855–3867. doi: 10.1016/j.neuropsychologia.2010.09.018, PMID: 20875439 PMC3712783

[ref33] Nobusako S. Ishibashi R. Takamura Y. Oda E. Tanigashira Y. Kouno M.. (2018). Distortion of visuo-motor temporal integration in apraxia: evidence from delayed visual feedback detection tasks and voxel-based lesion-symptom mapping. Front. Neurol. 9:709. doi: 10.3389/fneur.2018.00709, PMID: 30210434 PMC6119712

[ref34] Nobusako S. Osumi M. Hayashida K. Furukawa E. Nakai A. Maeda T.. (2020a). Altered sense of agency in children with developmental coordination disorder. Res. Dev. Disabil. 107:103794. doi: 10.1016/j.ridd.2020.103794, PMID: 33086140

[ref35] Nobusako S. Takamura Y. Koge K. Osumi M. Maeda T. Morioka M. (2024). Developmental changes in the time window for the explicit sense of agency experienced across the lifespan. Cogn. Dev. 72:101503. doi: 10.1016/j.cogdev.2024.101503

[ref36] Nobusako S. Tsujimoto T. Sakai A. Shuto T. Hashimoto Y. Furukawa E.. (2020b). The time window for sense of agency in school-age children is different from that in young adults. Cogn. Dev. 54:100891. doi: 10.1016/j.cogdev.2020.100891

[ref37] Ochipa C. Rapcsak S. Z. Maher L. M. Rothi L. J. Bowers D. Heilman K. M. (1997). Selective deficit of praxis imagery in ideomotor apraxia. Neurology 49, 474–480. doi: 10.1212/wnl.49.2.474, PMID: 9270580

[ref38] Oi H. Wen W. Chang A. Y. Uchida H. Maeda T. (2024). Hierarchical analysis of the sense of agency in schizophrenia: motor control, control detection, and self-attribution. Schizophr. (Heidelb.). 10:79. doi: 10.1038/s41537-024-00512-x, PMID: 39343773 PMC11439912

[ref39] Osiurak F. Lesourd M. Rossetti Y. Baumard J. (2019). Is there really a loss of agency in patients with apraxia of tool use? Front. Psychol. 10:87. doi: 10.3389/fpsyg.2019.00087, PMID: 30804829 PMC6370719

[ref40] Osiurak F. Reynaud E. Baumard J. Rossetti Y. Bartolo A. Lesourd M. (2021). Pantomime of tool use: looking beyond apraxia. Brain Commun. 3:fcab263. doi: 10.1093/braincomms/fcab263, PMID: 35350708 PMC8936430

[ref41] Osiurak F. Rossetti Y. (2017). Definition: Limb apraxia. Cortex 93:228. doi: 10.1016/j.cortex.2017.03.010, PMID: 28410626

[ref42] Osumi M. Nobusako S. Zama T. Yokotani N. Shimada S. Maeda T.. (2019). The relationship and difference between delay detection ability and judgment of sense of agency. PLoS One 14:e0219222. doi: 10.1371/journal.pone.0219222, PMID: 31287829 PMC6615602

[ref43] Pazzaglia M. (2022). The role of body in brain plasticity. Brain Sci. 12:277. doi: 10.3390/brainsci12020277, PMID: 35204040 PMC8869932

[ref44] Pazzaglia M. Galli G. (2014). Loss of agency in apraxia. Front. Hum. Neurosci. 8:751. doi: 10.3389/fnhum.2014.00751, PMID: 25295000 PMC4172088

[ref45] Pazzaglia M. Galli G. (2015). Translating novel findings of perceptual-motor codes into the neuro-rehabilitation of movement disorders. Front. Behav. Neurosci. 9:222. doi: 10.3389/fnbeh.2015.00222, PMID: 26347631 PMC4543860

[ref46] Pazzaglia M. Galli G. (2019). Action observation for neurorehabilitation in apraxia. Front. Neurol. 10:309. doi: 10.3389/fneur.2019.00309, PMID: 31001194 PMC6456663

[ref47] Ratcliffe N. Newport R. (2017). The effect of visual, spatial and temporal manipulations on embodiment and action. Front. Hum. Neurosci. 11:227. doi: 10.3389/fnhum.2017.00227, PMID: 28522968 PMC5415570

[ref48] Shimada S. Qi Y. Hiraki K. (2010). Detection of visual feedback delay in active and passive self-body movements. Exp. Brain Res. 201, 359–364. doi: 10.1007/s00221-009-2028-6, PMID: 19830411

[ref49] Sirigu A. Daprati E. Ciancia S. Giraux P. Nighoghossian N. Posada A.. (2004). Altered awareness of voluntary action after damage to the parietal cortex. Nat. Neurosci. 7, 80–84. doi: 10.1038/nn1160, PMID: 14647290

[ref50] Sirigu A. Duhamel J. R. (2001). Motor and visual imagery as two complementary but neurally dissociable mental processes. J. Cogn. Neurosci. 13, 910–919. doi: 10.1162/089892901753165827, PMID: 11595094

[ref51] Sirigu A. Duhamel J. R. Cohen L. Pillon B. Dubois B. Agid Y. (1996). The mental representation of hand movements after parietal cortex damage. Science 273, 1564–1568. doi: 10.1126/science.273.5281.1564, PMID: 8703221

[ref52] Synofzik M. Vosgerau G. Newen A. (2008a). Beyond the comparator model: a multifactorial two-step account of agency. Conscious. Cogn. 17, 219–239. doi: 10.1016/j.concog.2007.03.010, PMID: 17482480

[ref53] Synofzik M. Vosgerau G. Newen A. (2008b). I move, therefore I am: a new theoretical framework to investigate agency and ownership. Conscious. Cogn. 17, 411–424. doi: 10.1016/j.concog.2008.03.008, PMID: 18411059

[ref54] Synofzik M. Vosgerau G. Voss M. (2013). The experience of agency: an interplay between prediction and postdiction. Front. Psychol. 4:127. doi: 10.3389/fpsyg.2013.00127, PMID: 23508565 PMC3597983

[ref55] Tomasino B. Rumiati R. I. Umiltà C. A. (2003). Selective deficit of motor imagery as tapped by a left-right decision of visually presented hands. Brain Cogn. 53, 376–380. doi: 10.1016/s0278-2626(03)00147-7, PMID: 14607185

[ref56] Vanbellingen T. Kersten B. Van de Winckel A. Bellion M. Baronti F. Müri R.. (2011). A new bedside test of gestures in stroke: the apraxia screen of TULIA (AST). J. Neurol. Neurosurg. Psychiatry 82, 389–392. doi: 10.1136/jnnp.2010.213371, PMID: 20935324

[ref57] Vanbellingen T. Kersten B. Van Hemelrijk B. Van de Winckel A. Bertschi M. Müri R.. (2010). Comprehensive assessment of gesture production: a new test of upper limb apraxia (TULIA). Eur. J. Neurol. 17, 59–66. doi: 10.1111/j.1468-1331.2009.02741.x, PMID: 19614961

[ref58] Wolpe N. Moore J. W. Rae C. L. Rittman T. Altena E. Haggard P.. (2014). The medial frontal-prefrontal network for altered awareness and control of action in corticobasal syndrome. Brain 137, 208–220. doi: 10.1093/brain/awt302, PMID: 24293266 PMC3891444

[ref59] Wolpert D. M. Ghahramani Z. Jordan M. I. (1995). An internal model for sensorimotor integration. Science 269, 1880–1882. doi: 10.1126/science.7569931, PMID: 7569931

