# Supplementary material for: Distorted time window for sensorimotor integration and preserved time window for sense of agency in patients with post-stroke limb apraxia
Source: Front Hum Neurosci. 2025 Jun 13;19:1597200. doi: 10.3389/fnhum.2025.1597200 (PMC12202472; doi:10.3389/fnhum.2025.1597200)
Supplement: Supplementary file 1 [file Supplementary_file_1.docx]

**Supplementary Material. Detailed demographic and clinical characteristics of the apraxia and non-apraxia groups.**

| Group | Age (years) | Sex | Handedness | Disease | Lesion | Disease duration  (days) | MMSE | Kohs | Apraxia | | |  | Left upper limb function | |
| --- | --- | --- | --- | --- | --- | --- | --- | --- | --- | --- | --- | --- | --- | --- |
|  |  |  |  |  |  |  |  |  | Imitation | Gesture | Total score |  | Motor  function | Sensory function |
| Non-apraxia group (n = 11) | 71 | F | R | CH | Thalamus; Corona radiata; Crus posterius | 66 | 29 | 91.8 | 7 | 5 | 12 |  | 10 | 6 |
|  | 81 | F | R | CI | Corona radiata | 8 | 26 | 59.3 | 7 | 4 | 11 |  | 10 | 6 |
|  | 83 | F | R | CI | Corona radiata | 18 | 25 | 50 | 7 | 5 | 12 |  | 10 | 6 |
|  | 81 | M | R | CI | Medulla oblongata | 26 | 26 | 63.5 | 7 | 5 | 12 |  | 10 | 6 |
|  | 73 | M | R | CI | Corona radiata | 30 | 29 | 73 | 7 | 5 | 12 |  | 10 | 6 |
|  | 48 | F | R | CH | Subcortical frontal-parietal lobes | 108 | 29 | 77 | 7 | 4 | 11 |  | 10 | 6 |
|  | 51 | F | R | CT | Temporal lobe | 33 | 27 | 80.7 | 7 | 5 | 12 |  | 10 | 6 |
|  | 72 | F | R | CI | Corona radiata | 24 | 26 | 85.9 | 7 | 5 | 12 |  | 10 | 6 |
|  | 60 | M | R | CI | Postcentral gyrus; supramarginal gyrus | 88 | 28 | 82.5 | 7 | 5 | 12 |  | 10 | 6 |
|  | 55 | M | R | CH | Putamen | 37 | 28 | 98.4 | 7 | 5 | 12 |  | 10 | 6 |
|  | 68 | F | R | CI | Corona radiata | 62 | 29 | 98.4 | 7 | 5 | 12 |  | 10 | 6 |
| Mean | 67.5 | M, n = 4 F, n = 7 | R, n = 11 L, n = 0 | CI, n = 7 CH, n = 3 CT, n = 1 |  | 45.5 | 27.5 | 78.2 | 7.0 | 4.8 | 11.8 |  | 10.0 | 6.0 |
| SD | 11.8 |  |  |  |  | 30.0 | 1.4 | 15.0 | 0.0 | 0.4 | 0.4 |  | 0.0 | 0.0 |
| Minimum | 48 |  |  |  |  | 8 | 25 | 50 | 7 | 4 | 11 |  | 10 | 6 |
| Maximum | 83 |  |  |  |  | 108 | 29 | 98.4 | 7 | 5 | 12 |  | 10 | 6 |
| Skewness | -0.307 |  |  |  |  | 0.805 | -0.276 | -0.353 |  | -1.650 | -1.650 |  |  |  |
| Kurtosis | -1.257 |  |  |  |  | -0.069 | -1.584 | -0.655 |  | 2.037 | 2.037 |  |  |  |
| Apraxia group (n = 9) | 64 | F | R | CH | Putamen | 33 | 22 | 64 | 5 | 3 | 8 |  | 10 | 6 |
|  | 73 | F | R | CI | Thalamus | 15 | 25 | 93.2 | 5 | 3 | 8 |  | 10 | 6 |
|  | 51 | M | R | CH | Putamen | 50 | 30 | 94.7 | 5 | 3 | 8 |  | 10 | 6 |
|  | 72 | M | R | CH | Crus posterius; Corona radiata | 27 | 25 | 72.9 | 6 | 1 | 7 |  | 10 | 6 |
|  | 74 | F | R | CH | Putamen | 72 | 26 | 59.4 | 3 | 4 | 7 |  | 10 | 6 |
|  | 72 | M | R | CH | Crus posterius; Corona radiata | 94 | 25 | 72.9 | 5 | 3 | 8 |  | 10 | 6 |
|  | 58 | M | R | CH | Frontal-parietal cortex | 136 | 24 | 96 | 5 | 2 | 7 |  | 10 | 6 |
|  | 51 | F | R | CH | Putamen | 87 | 28 | 79 | 6 | 2 | 8 |  | 10 | 6 |
|  | 82 | F | R | CH | Thalamus; Crus posterius | 53 | 26 | 56.3 | 6 | 2 | 8 |  | 10 | 6 |
| Mean | 66.3 | M, n = 4 F, n = 5 | R, n = 9 L, n = 0 | CI, n = 1 CH, n = 8 CT, n = 0 |  | 63.0 | 25.7 | 76.5 | 5.1 | 2.6 | 7.7 |  | 10.0 | 6.0 |
| SD | 10.3 |  |  |  |  | 36.1 | 2.2 | 14.5 | 0.9 | 0.8 | 0.5 |  | 0.0 | 0.0 |
| Minimum | 51 |  |  |  |  | 15 | 22 | 56.3 | 3 | 1 | 7 |  | 10 | 6 |
| Maximum | 82 |  |  |  |  | 136 | 30 | 96 | 7 | 4 | 10 |  | 10 | 6 |
| Skewness | -0.288 |  |  |  |  | 0.582 | 0.434 | 0.106 | -1.213 | -0.177 | -0.707 |  |  |  |
| Kurtosis | -1.151 |  |  |  |  | 0.111 | 0.981 | -1.615 | 3.281 | 0.144 | -1.714 |  |  |  |

All lesions are in the left hemisphere.

CH, Cerebral hemorrhage; CI, Cerebral infarction; CT, Cerebral trauma; F, female; Kohs, Kohs block design Test; L, left; M, male; MMSE, mini mental state examination; R, right; SD, standard deviation.
